# Gas-Oxygen Anesthesia. Indications for Its Use

**Published:** 1914-07-15

**Authors:** A. H. Miller

**Affiliations:** Providence


### GAS-OXYGEN ANESTHESIA. INDICATIONS FOR ITS USE.

BY A. H. MILLER, M. D., PROVIDENCE.

There are a number of surgeons who hold that nitrous oxid and oxygen is the ideal anesthetic, and that it should be used for all surgical work. There is a larger group who maintain that this anesthetic has no place in the surgical field. In favor of the latter view are the facts that a number of deaths have resulted from the use of nitrous oxid and oxygen, and that this anesthetic does not produce sufficient relaxation in the average case for the work of many surgeons. On the other hand, it may be said that many lives have been saved through its use which would have been forfeited under any other anesthetic. No observer who has had an extensive experience with nitrous oxid and oxygen can have failed to observe such cases.

The deaths under nitrous oxid and oxygen anesthesia which have been reported are set down in the following table:

| OBSERVER. | DEATH ASCRIBED TO— | |
|---|---|---|
| Teter | Shock and primary cardiac failure | 1 |
| Crile | Myocarditis, six hours after operation | 2 |
| Lydston | Anesthetic | 3 |
| Allen | Uremia | 4 |
| Allen | No details | 25 |
| Allen | No details | 25 |
| Gatch | Hyperthyroidism | 5 |
| Gatch | Pericardial effusion | 5 |
| Gatch | Lymphatic diathesis | 5 |
| Olow | Diseased heart and arteries | 6 |
| Boys | Anesthetic | 7 |
| Miller | Suffocation (vomitus inspired) | 8 |
| Flagg | Anesthetic | 9 |
| Teter | Impure gas | 10 |
| Teter | Impure gas | 10 |
| Salzer | Anesthetic | 11 |
| Collins | Impure gas | 12 |
| Buchanan | Anesthetic | 13 |

The case reported by Olow in *Beitraege zur klinische Chirurgie* for December 1911, seems to have been reported as a separate case in *Surgery, Gynecology, and Obstetrics* for April 1912. Among the other cases to which Olow refers in the latter article as fatalities from nitrous oxid and oxygen anesthesia, that of Adams is included in Hewitt's list of seventeen deaths under nitrous oxid, not under gas-oxygen, and that of Owen was a death under nitrous oxid administered for a dental operation and not under nitrous oxid and oxygen anesthesia.

In a number of the foregoing cases, death occurred as a result of lack of care in the selection of the anesthetic. The nitrous oxid and oxygen was employed in spite of manifest contra-indications to its use. It is worth while to consider carefully the indications and contra-indications in the use of nitrous oxid and oxygen.

The indications for the use of nitrous oxid and oxygen anesthesia are as follows:

(1) Depending on the condition of the patient: Anemia; diabetes; acute infections; impaired kidneys, as nephritis; pyelitis; respiratory affections, especially in the aged, as tuberculosis, pneumonia, bronchitis, laryngitis; bad surgical risks, as in typhoid perforation; grave general peritonitis; intestinal obstruction; shock, collapse; debility, the very ill; old age; lowered vitality from any cause.

(2) Depending on the nature of the operation: Many minor operations, manipulations, examinations, and dressings; operations on extremities; obstetric operations; breast operations; operations for empyema; when the effect of surgical shock is feared.

(3) To avoid post-anesthetic nausea and vomiting.

The contra-indications to the use of nitrous oxid and oxygen anesthesia are as follows:

(1) Absence of expert anesthetist and perfected apparatus.

(2) Depending on the condition of the patient: Bad heart lesions with broken compensation, myocarditis, fatty heart, dilated heart; aneurysm; respiratory obstruction; enlarged tonsils and other marked narrowing of upper air-passages; tumors of neck, including thyroid growths; cellulitis of submaxillary and cervical region; emphysema; arterio-sclerosis; increased intracranial pressure from tumor or abscess; intestinal obstruction with distended abdomen; children under five years of age; strong, vigorous, rough men; extreme nervous temperament; addiction to drug habits, or use of tobacco or alcohol.

(3) Depending on the nature of the operation: When perfect relaxation is desired for operations on healthy subjects; operations on larnyx and trachea.

From the foregoing, the writer concludes that it is evident that nitrous oxid and oxygen is not the ideal anesthetic for use in all cases. It is also certain that it is an agent of the greatest value in certain selected cases. To determine the proper indications for its use, it is necessary to consider the condition of the patient and the nature of the operation. In no case should it be used in the absence of a skilled administrator.

Each one of Dr. Miller's statements is culled from and substantiated by one or several special articles on this subject which have appeared within recent years. His compilation, therefore, carries weight.—*N. Y. Med. Journal and Dental Cosmos.*

